# CT-based delta radiomics in predicting the prognosis of stage IV gastric cancer to immune checkpoint inhibitors

**DOI:** 10.3389/fonc.2022.1059874

**Published:** 2023-01-04

**Authors:** Jiazheng Li, Zifan Chen, Yang Chen, Jie Zhao, Meng He, Xiaoting Li, Li Zhang, Bin Dong, Xiaotian Zhang, Lei Tang, Lin Shen

**Affiliations:** ^1^ Department of Radiology, Key Laboratory of Carcinogenesis and Translational Research (Ministry of Education), Peking University Cancer Hospital and Institute, Beijing, China; ^2^ Center for Data Science, Peking University, Beijing, China; ^3^ Department of Gastrointestinal Oncology, Key Laboratory of Carcinogenesis and Translational Research (Ministry of Education), Peking University Cancer Hospital and Institute, Beijing, China; ^4^ National Engineering Laboratory for Big Data Analysis and Applications, Peking University, Beijing, China; ^5^ Beijing International Center for Mathematical Research (BICMR), Peking University, Beijing, China

**Keywords:** immunotherapy, gastric cancer, prognosis, radiomics, computed tomography

## Abstract

**Introduction:**

To explore the prognostic value of CT-based delta radiomics in predicting the prognosis of patients with stage IV gastric cancer treated with immune checkpoint inhibitors (ICI).

**Materials and methods:**

Forty-two patients with stage IV gastric cancer, who had received ICI monotherapy, were enrolled in this retrospective study. Baseline and first follow-up CT scans were analyzed. Intratumoral and peritumoral regions of interest (ROI) were contoured, enabling the extraction of 192 features from each ROI. The intraclass correlation coefficients were used to select features with high stability. The least absolute shrinkage and selection operator was used to select features with high weights for predicting patient prognosis. Kaplan–Meier analysis and log-rank test were performed to explore the association between features and progression free survival (PFS). Cox regression analyses were used to identify predictors for PFS. The C-index was used to assess the prediction performance of features.

**Results:**

Two radiomics features of ΔVintra_ZV and postVperi_Sphericity were identified from intratumoral and peritumoral regions, respectively. The Kaplan–Meier analysis revealed significant differences in PFS between patients with low and high feature value (ΔVintra_ZV: *P*=0.000; postVperi_Sphericity: *P*=0.012), and the multivariable cox analysis demonstrated that ΔVintra_ZV was independent predictor for PFS (HR, 1.911; 95% CI: 1.163–3.142; *P*=0.011), with C-index of 0.705.

**Conclusions:**

Based on CT scans at baseline and first follow-up, the delta radiomics features could efficiently predict the PFS of gastric cancer patients treated with ICI therapy.

## Introduction

Gastric cancer is one of the malignancies with high mortality rate (1). Despite significant efforts to develop innovative treatment techniques based on cytotoxic chemotherapy, targeted therapy, and radiotherapy, a significant proportion of gastric cancer patients will still demonstrate poor response to conventional therapies or even fast progression after treatment (2). Immune checkpoint inhibitors (ICIs) have revolutionized the treatment of a variety of malignancies, including gastric cancer (3). More specifically, several large multicenter clinical trials demonstrated a significant and durable survival benefit in refractory gastric cancer patients who received ICI therapy, with a duration of response ranging from 8.4 to 9.5 months (3, 4). However, treatment response varied significantly as 60% of patients derived no benefit from ICI therapy, and 21% of patients even showed hyperprogression during treatment (2, 3, 5). Therefore, there is an urgent need for the introduction of precise biomarkers that can predict the response of gastric cancer to ICI at the early treatment stage.

Several predictive tumor biomarkers from biopsy tissue samples could indicate ICI treatment response and prognosis of gastric cancer patients (6), such as positivity of programmed death-ligand 1 (PD-L1), mismatch repair deficiency (dMMR), and Epstein–Barr virus (EBV) (6, 7). However, most patients tested negative for the above-mentioned biomarkers, e.g., 86% of patients with PD-L1 combined positive scores (CPS)<1, 78.4-92.5% of patients with mismatch repair proficiency (pMMR), and 91% of patients with negative EBV according to previous studies (3, 5, 8, 9). Some of these biomarkers predicted that patients with poor treatment response could still respond well to ICI therapy, with reported objective response rates of approximately 6.4-10.9% for PD-L1 CPS < 1, 12.3% for pMMR, and 16.4% for negative EBV, respectively (3, 6, 7). Besides, not all laboratories have the available resources to perform complex immunohistochemistry protocols that are necessary to identify or evaluate potential tumor biomarkers, hindering their subsequent application in clinical practice (10, 11). In addition, given the spatial heterogeneity of gastric cancer, biopsy samples may not always be evaluated appropriately.

Computed tomography (CT) has been widely and routinely used in clinical practice, yet traditional unidimensional measurements made both RECIST and iRECIST criteria no longer meet the needs of the ICI response evaluation and hindered the realization of the precision medicine (12). Radiomics is a useful tool to mine data from radiographic images, such as tumor texture characteristics, which may not be detectable by ‘naked-eye’ inspection (13). Several studies have verified that radiomics could predict response to neoadjuvant chemotherapy and palliative chemotherapy in patients with gastric cancer, with an area under the curve (AUC) of 0.74-0.82 (14–16). Recently, one study explored the response prediction performance of baseline CT radiomics in patients treated with immunotherapy combined with chemotherapy and showed promising results, with an AUC over 0.7 (17). To the best of our knowledge, the prognostic value of radiomics features in patients with gastric cancer treated with ICI monotherapy has not been elucidated. Therefore, this study aimed to use delta radiomics to extract information from CT scans (at baseline and first follow-up) and predict the survival of patients with stage IV gastric cancer treated by ICI.

## Materials and methods

### Patients

**Figure 1:**
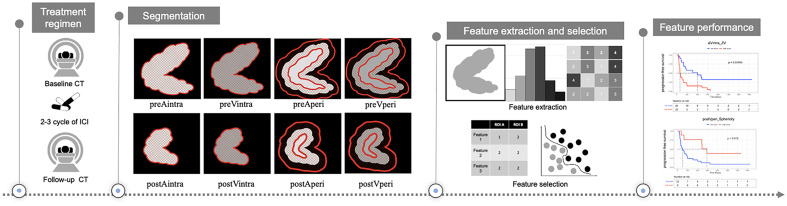
Study design for the evaluation of response prediction to immune checkpoint inhibitors in stage IV gastric cancer patients based on radiomics features. preAintra: intratumoral regions on baseline atrial phase; preVintra: intratumoral regions on baseline venous phase, preAperi: peritumoral regions on baseline atrial phase; preVperi: peritumoral regions on baseline venous phase; postAintra: intratumoral regions on follow-up atrial phase; postVintra: intratumoral regions on follow-up venous phase; postAperi: peritumoral regions on follow-up atrial phase; postVperi: peritumoral regions on follow-up venous phase.

This study was performed in line with the principles of the Declaration of Helsinki. Written informed consent of this retrospective study was waived. Data from 101 consecutive patients with stage IV gastric cancer who had received anti-programmed cell death protein 1/programmed cell death ligand 1 (PD-1/PD-L1) antibody alone or in combination with anti-cytotoxic T lymphocyte-associated antigen 4 (CTLA-4) antibody were collected in the Peking University Cancer Hospital, Beijing, China, between 2016 and 2020. Inclusion criteria were as follows: (a) histologically confirmed gastric adenocarcinoma; (b) patients treated with ICI monotherapy (anti-PD-1/PD-L1 alone or in combination with anti-CTLA-4 antibodies); (c) availability of baseline enhanced abdominal/pelvic CT scans performed <30 days before ICI treatment; (d) availability of the first follow-up enhanced abdominal/pelvic CT scans two to three cycles after ICI treatment initiation. Exclusion criteria were as follows: (a) patients with primary gastric surgical treatment (n=53) (b) patients with other synchronous or metachronous malignant neoplasms (n=4); (c) thickness of primary gastric lesions <10mm on CT (n=1); (d) CT images with obvious artifacts (n=1). Finally, 42 patients were included in our study. The following clinicopathological data were retrospectively collected from patients’ medical records: age, gender, Eastern Cooperative Oncology Group performance status score (ECOG PS), treatment regimen, treatment cycles of ICI before first follow-up, Lauren subtype, degree of differentiation, PD-L1 status, MMR status, EBV status, peritoneal metastasis, hepatic metastasis, the number of metastatic sites. We registered patients with PD-L1 CPS ≥ 1 as PD-L1 positive cases (4). The flowchart is shown in **Figure 1**.

### Treatment regimens and follow-up protocol

There were 29 patients received anti-PD-1/PD-L1 treatment alone, including seven patients received GLS-010 (zimberelimab) (240mg d1 d15 Q28d), six patients received CS1003 (nofazinlimab) (200mg d1 Q21d), four patients received toripalimab (3mg/kg d1 d15 Q28d), three patients received BGB-A317 (tislelizumab) (200mg d1 Q21d), three patients received pembrolizumab (200mg d1 Q14d), two patients received atezolizumab (1200mg d1 Q21d), two patients received MSB2311 (20mg/kg d1 Q21d), one patients received LZM009 (432mg d1 Q28d) and one patients received Sintilimab (200mg d1 Q21d). There were 13 patients received anti-PD-1 in combination with anti-CTLA-4 treatment, including seven patients received Sintilimab + IBI310 (Sintilimab 200mg d1 Q21d, IBI310 68mg d1 Q42d) and six patients received Nivolumab + Ipilimumab (Nivolumab 1mg/kg d1, d22 Q42d, Ipilimumab 3mg/kg d1 d22 Q42d).

All patients conducted follow-up every two to three cycles of ICI treatment, including enhanced abdominal/pelvic CT scans until the resistance to ICI therapy. PFS was defined as the time from the start of ICI treatment to disease progression, death from any cause, or the cutoff date of November 12, 2021. Patients without any progression or death at the end of the follow-up period were censored.

### CT examination

All patients underwent abdominal/pelvic contrast-enhanced CT examinations after fasting for more than eight hours. 10 mg anisodamine (654-2, Hangzhou Minsheng Pharma) was administered intramuscularly to reduce gastrointestinal motility before CT examination. Next, 6g gas-producing crystals with 10ml warm water were given orally shortly before the examination. All patients underwent a quick respiratory training session to prevent potential respiratory artifacts. The CT scanner was either the LightSpeed 64 VCT or the Discovery CT750 HD, with a peak tube voltage of 120 kVp, an automatic tube current-time product, a collimation thickness of 64 x 0.625 mm, a helical pitch of 0.984:1, 5-mm scanning thickness, and 0.625-mm reconstructed thickness. Patients were scanned in the supine position, and scan coverage started from the diaphragmatic dome until 2cm below the lower margin of symphysis ossium pubis. All patients were injected with nonionic contrast material through the antecubital vein at a rate of 3.5ml/s (1.5ml/kg of body weight, iohexol 300mg I/ml, Omnipaque, GE Healthcare). Arterial and venous phase scanning were performed at 40s and 70s, respectively, following contrast media injection.

### Image analysis and segmentation

**Figure 2:**
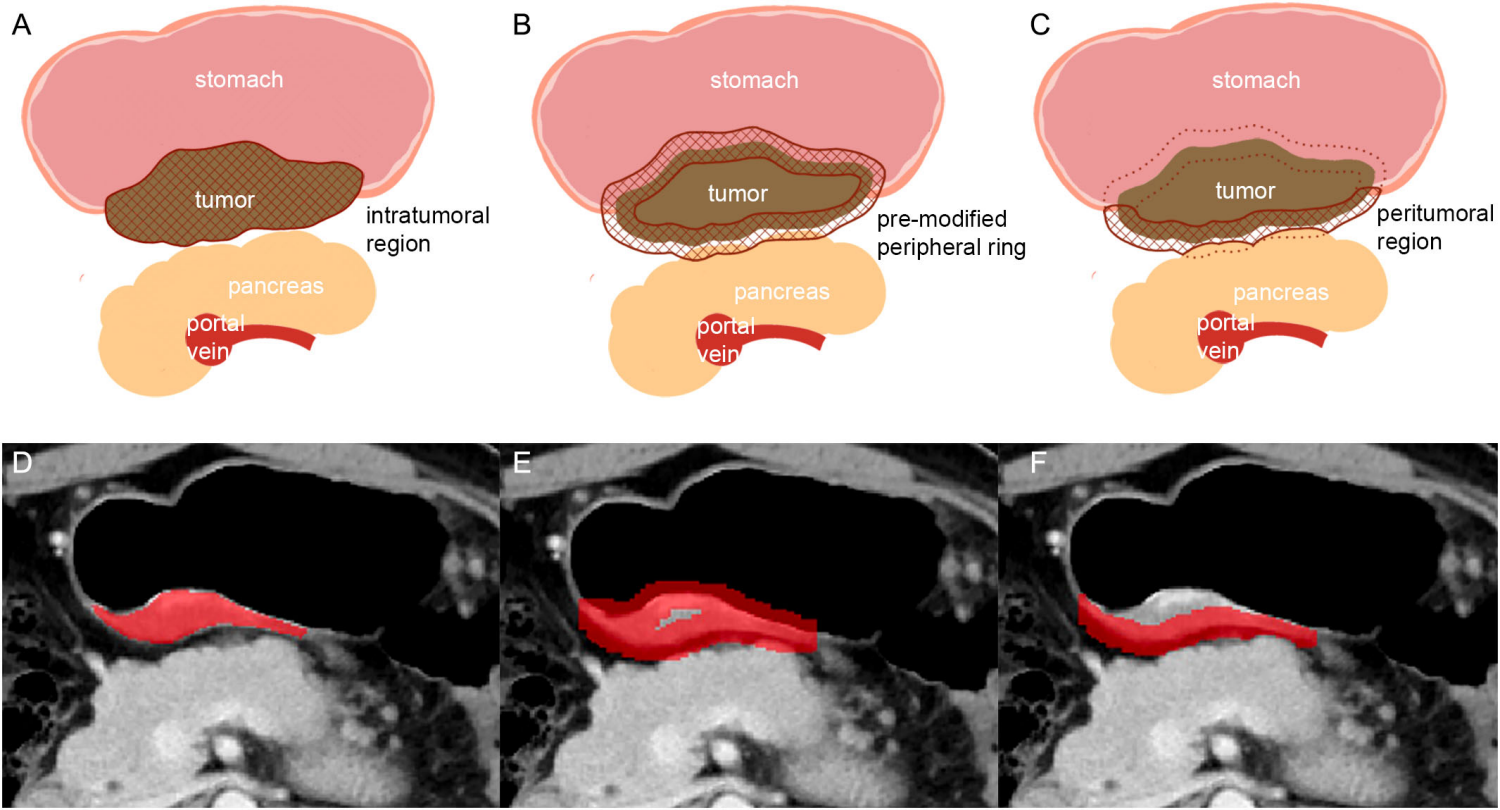
Schematic illustration of the steps followed to draw intratumoral and peritumoral ROIs. **(A, D)**: the largest axial sections of gastric lesions were manually contoured (intratumoral region of ROI). **(B, E)**: the pre-modified peripheral ring was automatically generated. **(C, F)**: modifying was conducted manually on the pre-modified peripheral ring to exclude the gastric cavity and the part covering the surrounding organs (pancreas) and large vessels (in C: solid line: peritumoral region of ROI; dotted line: the part of ROI been deleted).

Baseline and first follow-up CT scans in arterial and venous phases were analyzed by two radiologists with 20 and 3 years of experience in gastrointestinal CT interpretation, respectively (TL and LJZ). Both radiologists were blinded to the clinical and histopathological information. However, they did know the anatomical location of gastric cancer. Two intratumoral regions of interest (ROI) were manually contoured–one ROI for the arterial phase and another ROI for the venous phase–on the largest area of the gastric lesions (axial plane) using the ITK-SNAP software (v.3.6.0, http://www.itksnap.org). To capture peritumoral information, the slice image was uniformly interpolated to 0.6 mm per pixel, and a peripheral ring was then created automatically by dilating the tumor boundaries by 7 pixels (4.2mm) on the outside and shrinking by 7 pixels (4.2mm) on the inside (18). Secondly, the modification was conducted manually on the pre-modified peripheral ring to exclude the gastric cavity and the area covering the surrounding organs and large vessels (**Figure 2**).

### Feature extraction and selection

First, we uniformly resampled the CT image and its ROI annotation so that the spacing parameters in the x-, y-, and z-axis were 0.6, 0.6, and 5.0, respectively. The texture features were extracted from each ROI of each patient’s CT image using the open-source python platform Pyradiomics (version 2.1.2, https://pyradiomics.readthedocs.io/en/latest/#). We extracted a total of 192 features for each ROI, including eight shape features, 36 first-order statistics, 46 gray level co-occurrence matrices, 32 gray level run length matrices, 32 gray level size zone matrices, 28 gray level dependence matrices, and ten neighboring gray tone difference matrices. Eight sets of radiomics features were derived from intratumoral and peritumoral regions at baseline arterial and venous phases, and follow-up CT scans, including features from intratumoral regions at baseline atrial phase (preAintra), intratumoral regions at baseline venous phase (preVintra), peritumoral regions at baseline atrial phase (preAperi), peritumoral regions at baseline venous phase (preVperi), intratumoral regions at follow-up atrial phase (postAintra), intratumoral regions at follow-up venous phase (postVintra), peritumoral regions at follow-up atrial phase (postAperi), and peritumoral regions at follow-up venous phase (postVperi). Calculate the changes between baseline and follow-up features by subtracting the values of CT features of follow-up and baseline, which provided four corresponding sets of delta features (ΔAintra, ΔVintra, ΔAperi, and ΔVperi).

All radiomics features were standardized by subtracting the mean value and dividing by the standard deviation. Intraclass correlation coefficients (ICCs) based on a multiple-raters, two-way random-effects model were calculated to assess the stability and reproducibility of radiomic features within groups. To ensure reliability for all twelve sets of radiomics features, we only reserved radiomic features with ICC estimates > 0.80, and further selection was then conducted in the data obtained by TL. Furthermore, we used the Cox proportional hazards regression method with the least absolute shrinkage and the selection operator (LASSO) penalty with four-fold cross-validation to select the most useful predictive features from intratumoral and peritumoral regions, respectively (19). Since the total patient number was limited, the most significant nonzero feature in intratumoral and peritumoral regions was selected to avoid overfitting.

### Statistical analysis

Continuous variables were presented as the mean with standard deviation (SD) or median with interquartile ranges (IQR) based on normal distribution or not. Categorical variables were shown as numbers with percentages. PFS was estimated using the Kaplan–Meier method, and the log-rank test was employed to compare differences in survival probability. The Cox proportional hazards model was used for univariate and multivariate analyses. *P* values less than 0.10 in univariate analysis were subsequently included in the multivariate analyses where enter feature selection was used. Harrell’s concordance index (C-index) was calculated to evaluate prognostic ability. Statistical analysis was conducted using R software (R 4.0.4, The R Foundation for Statistical Computing, Vienna, Austria). All statistical tests were two-sided, and a value of *P*<0.05 was considered significant.

## Results

### Patient characteristics

A total of 42 patients were included in this study. The median follow-up time and the median time for PFS were 736 (IQR: 656, 1266) and 133 (IQR: 61, 483) days, respectively. The patients’ clinicopathological data are summarized in **Table 1**. Univariate analysis revealed that age and Lauren type were associated with PFS. In contrast, other clinicopathological characteristics were not found to have a prognostic impact. The K–M analysis showed that older patients (>62 years, median value) had more prolonged PFS compared to younger patients [median PFS time: younger patients, 92 (IQR: 45, 165) days; older patients, 483 (IQR: 73, not reached) days; P=0.001]. Patients with intestinal-type gastric cancer showed more prolonged PFS than patients with a different Lauren type [median PFS time: intestinal type, 195 (IQR: 100, 649) days, reference; diffuse type, 63 (IQR: 45, 92) days, *P* =0.003; mixed type, 127 (IQR: 54, 134) days, *P*=0.087].

**Table 1:** The clinicopathological characteristics of the included patients.

Characteristics	Total (n=42)	Univariate analysis		Multivariate analysis		
		HR (95%CI)	*P* value	HR (95%CI)	*P* value
Age (years), (median [IQR])	62.00 (12.00)	0.943 (0.915–0.972)	0.000*	0.972 (0.936–1.008)	0.129
Gender, n (%)		0.534 (0.257–1.112)	0.094		
Male	31(73.814%)				
Female	11(26.19%)				
ECOG PS, n (%)		0.658 (0.353–1.223)	0.186		
0	20 (47.628%)				
1 +–2	22 (52.3852%)				
Treatment regimen, n (%)		0.797 (0.359–1.769)	0.576		
Anti-PD-1/PD-L1	29 (69.04%)				
Anti-PD-1 + anti-CTLA-4	13 (30.95%)				
Treatment cycle, n (%)		1.229 (0.820–1.844)	0.318		
Two cycles	33 (78.57%)				
Three cycles	9 (21.43%)				
Lauren type, n (%)					
Intestinal	19 (45.23%)	[reference]		[reference]	
Diffuse	11 (26.19%)	3.629 (1.553–8.478)	0.003*	3.155 (1.203–8.275)	0.020*
Mixed	7 (16.677%)	2.370 (0.883–6.362)	0.087	1.924 (0.668–5.540)	0.225
No testing	5 (11.9012%)	0.570 (0.161–2.026)	0.385	0.409 (0.087–1.933)	0.259
Differentiation, n (%)		1.157 (0.578–2.317)	0.680		
Moderate	17 (40.481%)				
Poor	25 (59.5260%)				
PD-L1, n (%)					
Negative	12 (28.579%)	[reference]			
Positive	17 (40.48%)	0.520 (0.228–1.185)	0.119		
No testing	13 (30.951%)	0.604 (0.256–1.427)	0.250		
MMR, n (%)					
pMMR	33 (78.579%)	[reference]			
dMMR	4 (9.5210%)	0.296 (0.069–1.279)	0.103		
No testing	5 (11.902%)	2.282 (0.836–6.227)	0.107		
EBV, n (%)					
Negative	23 (54.765%)	[reference]			
Positive	12 (28.579%)	0.875 (0.361–2.122)	0.768		
No testing	7 (16.677%)	2.103 (0.834–5.310)	0.115		
peritoneal metastasis, n (%)		0.513 (0.254–1.036)	0.063	1.187 (0.469–3.007)	0.717
Present	20 (47.62%)				
Absent	22 (52.38%)				
Hepatic metastasis, n (%)		1.263 (0.616–2.589)	0.524		
Present	15 (35.71%)				
Absent	27 (64.29%)				
Number of metastatic sites, n (%)					
1	7 (16.67%)	[reference]		[reference]	
2	29 (69.05%)	1.640 (0.566–4.755)	0.362	2.022 (0.546–7.481)	0.292
3+	6 (14.29%)	3.431 (0.955–12.321)	0.059	4.881 (0.884–26.939)	0.069
ΔVintra_ZV, (median [IQR])	-0.07 (0.54)	2.320 (1.478 – 3.641)	0.000*	1.911 (1.163–3.142)	0.011*
postVperi_Sphericity, mean (SD)	0.00 (1.00)	0.601 (0.410 – 0.882)	0.009*	0.690 (0.421–1.132)	0.142

IQR, interquartile ranges; HR, hazard ratio; CI, confidence interval; ECOG PS, Eastern Cooperative Oncology Group performance status score; PD-1, programmed cell death protein 1; PD-L1, programmed cell death ligand 1; MMR, mismatch repair; dMMR, mismatch repair deficiency; pMMR, mismatch repair proficiency; EBV, Epstein–Barr virus.

### Radiomics feature selection

A three-step radiomics feature selection procedure was applied. In the first step, 2304 radiomics features were extracted from twelve sets of features. Consequently, 99 features were further enrolled with ICC>0.80 as a reliability standard, including 70 intratumoral features (preAintra: 14; preVintra: 25; postAintra: 8; postVintra:13; ΔAintra: 2, ΔVintra: 8) and 29 peritumoral features (preAperi: 5; preVperi: 8; postAperi: 7; postVperi: 8; ΔAperi: 0, ΔVperi: 1). The third step involved the selection of features with the highest coefficient based on the Lasso COX method, which included ΔVintra_original_glszm_Zone Variance (ΔVintra_ZV) from the intratumoral regions and postVperi_original_shape_Sphericity (postVperi_Sphericity) from the peritumoral regions.

### Radiomics feature analysis

**Figure 3:**
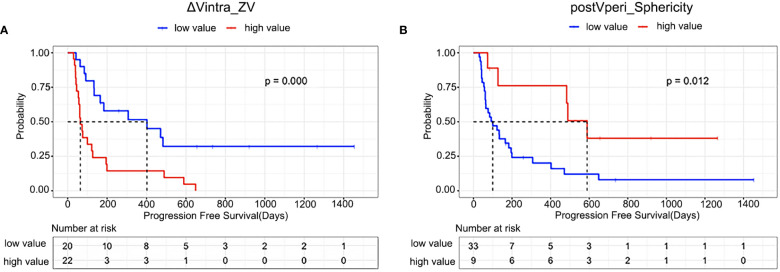
Kaplan–Meier estimates of progression-free survival in all patients according to **(A)** ΔVintra_ZV (ΔVintra_original_glszm_Zone Variance); **(B)** postVperi _Sphericity (postVperi_original_shape_Sphericity).

**Figure 4:**
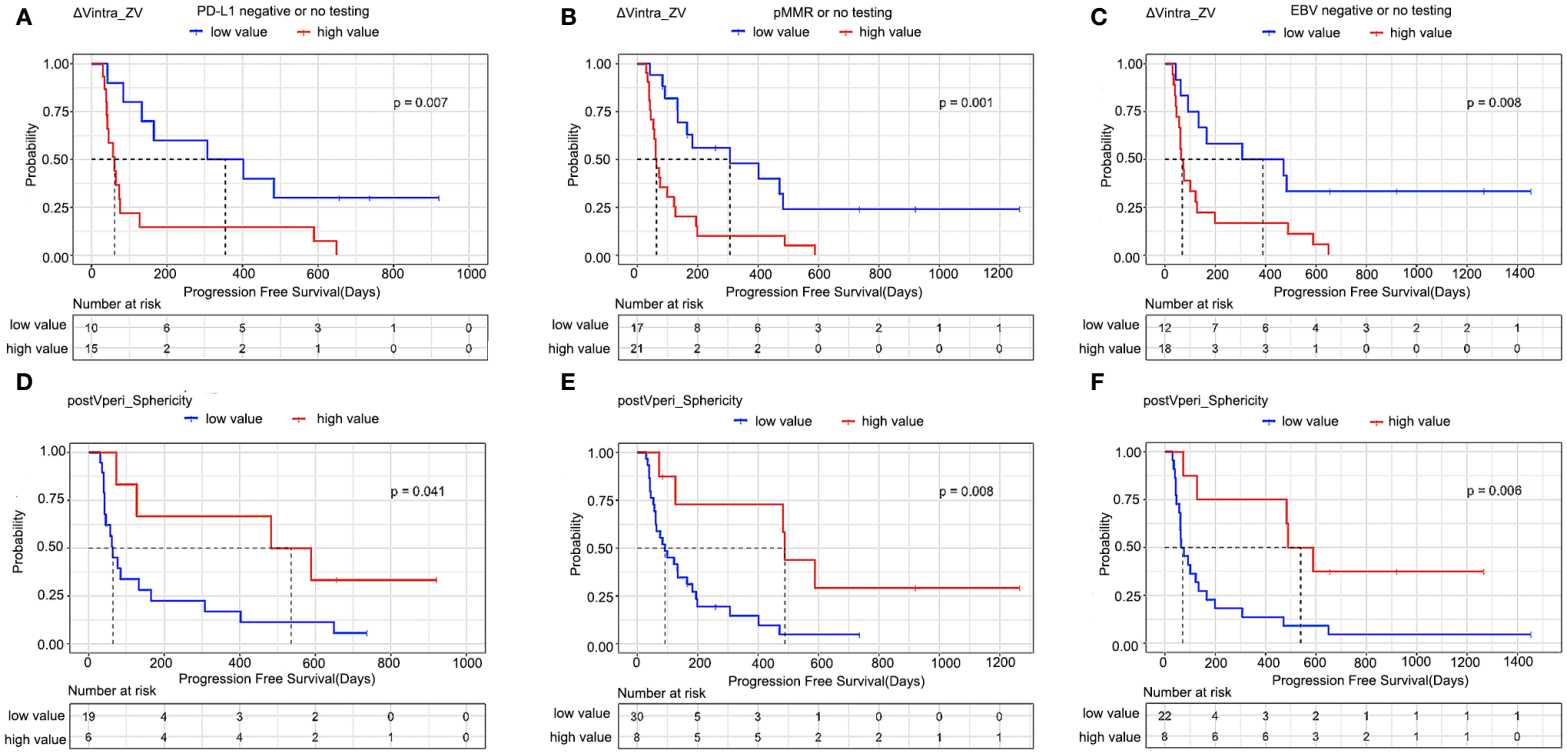
Kaplan–Meier estimates of progression-free survival in patients who had either not been tested or had already tested negative for biomarkers. **(A, D)** PD-L1, **(B, E)** MMR, and **(C, F)** EBV according to radiomics features. ΔVintra_ZV, ΔVintra_original_glszm_Zone Variance; postVperi_Sphericity, postVperi_original_shape_Sphericity.

The optimal cut-off values were -0.09 and 0.88 for ΔVintra_ZV and postVperi_Sphericity determined by X-tile (version 3.6.1), respectively. The K–M analysis suggested that the PFS of stage IV gastric cancer patients with a high ΔVintra_ZV value (> cutoff value) was worse than that of patients with a low value (≤ cutoff value), with a median PFS of 402 *vs*. 64 days (*P*=0.000, log-rank test). The PFS of stage IV gastric cancer patients with a low postVperi_Sphericity value was worse than that of patients with a high value, with a median PFS of 100 *vs*. 589 days (*P*=0.012, log-rank test) (**Figure 3**). We performed additional analyses within subgroups of gastric cancer patients who had either not been tested or had already tested negative for biomarkers, including PD-L1, MMR, and EBV. Our findings revealed that ΔVintra_ ZV and postVperi Sphericity could stratify patients in all three subgroups according to their PFS (**Figures 4A–F**).

ΔVintra_ZV and postVperi_Sphericity were both significant in univariate analysis (hazard ratio [HR], 2.320; 95% confidence interval [CI]: 1.478–3.641, *P*=0.000; HR, 0.601; 95% CI: 0.410–0.882, *P*=0.009). After controlling for age, Lauren type, peritoneal metastasis, and number of metastatic sites, ΔVintra_ZV were still independent predictor of survival (HR, 1.911; 95% CI: 1.163–3.142; *P*=0.011). However, postVperi_Sphericity had no association with PFS (HR, 0.690; 95% CI: 0.421–1.132; *P*=0.142). ΔVintra_ ZV and postVperi_Sphericity yielded a C-index of 0.705 (95% CI: 0.625–0.785) and 0.632 (95% CI: 0.528–0.736), respectively.

## Discussion

This study initially explored the relationship between delta radiomics with the prognosis of patients with stage IV gastric cancer receiving ICI. Our findings revealed that ΔVintra_ZV and postVperi_Sphericity from the intratumoral and peritumoral regions, respectively, could classified patients with survival outcomes and ΔVintra_ZV was the independent predictor for PFS.

Previous studies have reported that on-treated tumor samples of patients with effective ICI response showed increased immune cell abundance and a low percentage of tumor cells (20, 21). A previous study explored the association between radiomics features in pan-cancer and CD8 cell abundance within the tumor. The relatively homogeneous tumors were associated with increased pre-existing CD8+ cell infiltration and better prognosis; in contrast, tumors composed of highly proliferating tumor cells exhibited a more heterogeneous radiomics texture (22). In our study, the low ΔVintra_ZV score indicated that the texture of gastric lesions changed from heterogeneous to homogenous and thus were more likely to be observed in patients with prolonged survival after ICI treatment. We hypothesized that this change may indicate immune cell infiltration and good tumor response to ICI therapy, whereas texture changes towards non-uniformity may indicate a high extent of tumor cell proliferation and resistance to ICI. Similar to our results, Basler et al. also suggested that the changes in the CT texture of metastatic melanoma from heterogeneity to homogeneity during ICI treatment are more likely to represent pseudoprogression, whereas changes from homogeneity to heterogeneity may indicate true tumor progression (23). Accordingly, patients with pseudoprogression showed longer survival compared with patients with true progression (23).

Given the dynamic change of tumor-immune interactions, biomarkers capable of tracking tumor evolution during the treatment course may provide more information on patients’ prognoses. A previous histological study showed that early on-treatment samples were more predictive of the response to ICI compared to the mere assessment of baseline samples (20). Although biopsies provide a method to capture the dynamic change of tumors, invasive re-biopsy may not be frequently conducted in real-world clinical practice. In our study, both predictors, ΔVintra_ ZV and postVperi_ Sphericity, incorporated post-treatment CT texture features and could predict the response of patients receiving ICI. Consistently, Khorrami et al. developed radiomics models to predict the ICI response and OS of patients with non–small cell lung cancer (NSCLC). The results showed that the performance of models combining baseline and follow-up features was better than the baseline radiomics model alone (21). Similar results also have been reported in patients treated with radiation therapy and chemotherapy (24, 25).

Khorrami et al. have shown that the ICI response prediction performance of combined radiomics from intra- and peritumoral regions in NSCLC was superior to radiomics from the intratumoral region alone (21). The authors also found that the density of immune infiltration in surgical specimens after ICI was correlated with peritumoral delta radiomics (21). In previous articles, the association between peritumoral radiomics and pathological characteristics of gastric cancer were also studied, but the prediction value of peritumoral radiomics were different (18, 26, 27). Some large-scale studies showed peritumoral radiomics features were one of the important factors to determine the tumor immune microenvironment of gastric cancer and had the prognosis predicting value, while another large-scale study showed peritumoral features may be inapplicable for predicting the Lauren classification of gastric cancer (18, 26, 27). We noticed that the peritumoral ROI in their studies were all a peripheral ring, the same with the peritumoral ROI used in lung cancer (18, 21, 26, 27). However, unlike lung cancer which is surrounded by consistent pulmonary tissue, gastric cancer is usually surrounded by air in the stomach cavity, fat tissue of peritoneum and adjacent organs. We suppose the peritumoral ROI covering air, fat, gastric cancer, and even other organs may influence the precision of information from radiomics features of the peritumoral region, although thickness of ROI around the tumor used in previous studies were smaller than ours. Therefore, in our research, we put effort into modifying the automatically generated peripheral ring, especially deleting the adjacent organs and air covered by the automatically generated ROI. However, unfortunately, such procedure increased the interobserver variability, and only 29 peritumoral features had ICC > 0.80 (70 intratumoral features had ICC > 0.80). Moreover, it was labor-intense to modify the peritumoral ROI of all patients. Taken together, we believe that further research is needed to explore the appropriate method for extracting information from the peritumoral region of gastric cancer.

In our study, patients with a high postVperi_Sphericity score demonstrated a trend towards a more promising survival outcome compared to patients with a low score. Sphericity measured the roundness of the shape and a larger value meant that the shape of ROI resembled a circle (28). Given that all ROIs of the peritumoral area appeared long and narrow, high score of postVperi_ Sphericity could be considered in two aspects, the larger the width and the shorter the length of the ROI. The width of the pre-modified peripheral ring was consistent among different patients (9.4mm in total) when first generated automatically. In patients with low visceral adipose tissue, the pre-modified peripheral ring may cover adjacent organs and thus should be manually modified, contributing to a smaller width. Poor nutritional status, including low visceral fat, has been associated with worse survival outcomes in patients treated with ICI therapy (29, 30). The length of ROI could be regarded as the maximum tumor extension on stomach. Maximum tumor diameter has been proved to be a negative factor for prognosis of patients with gastric cancer (31). Therefore, we considered that a low sphericity score may reflect poor nutritional status and high tumor burden and indicate worse survival after ICI treatment.

Our study has some limitations. First, the sample size of this retrospective study was relatively small. However, the data obtained from patients treated with ICI monotherapy were informative and of great value for assessing response after ICI treatment. In contrast, a combination regimen, such as ICI and chemotherapy, may cause confounding factors. Our study should be considered exploratory. Second, histological biomarker data were unavailable from all patients in this study. Since not all hospitals have accredited laboratories to carry out complex immunohistochemistry protocols, it is worthwhile to investigate the predictive value of radiomics features in patients who have not been tested or have already tested negative for biomarkers to provide a method of selecting appropriate treatment. Third, pathology confirmation of immune cell infiltration from post-treatment samples was absent. Future studies should aim to evaluate the relationship between radiomics features and immune cell infiltration in post-treatment gastric cancer samples.

## Conclusions

Given the complexity of the intrinsic biological pathway of the tumor microenvironment, current biomarkers alone, including PD-L1, dMMR, and EBV status, cannot predict patient prognosis completely. Radiomics features complement these widely accepted histological biomarkers and can be considered candidate biomarkers that can reflect tumor phenotype and provide longitudinal surveillance. Radiomics features have the potential to be used as cost-effective screening tools that can be applied in clinical practice when administering ICI treatment to patients with gastric cancer.

## Data availability statement

The raw data supporting the conclusions of this article will be made available by the authors, without undue reservation.

## Ethics statement

The studies involving human participants were reviewed and approved by Peking Cancer Hospital. The ethics committee waived the requirement of written informed consent for participation.

## Author contributions

JL and ZC contributed equally to this work and share first authorship; BD, XZ, LT, and LS contributed equally to this work and share corresponding authorship. All authors contributed to the article and approved the submitted version.

## Glossary

**Table:**

ICI	Immune checkpoint inhibitors
ROI	Regions of interest
PFS	Progression free survival
PD-1	programmed cell death protein 1
PD-L1	programmed cell death ligand 1
CTLA-4	Cytotoxic T lymphocyteassociated antigen 4
dMMR	Mismatch repair deficiency
EBV	Epstein–Barr virus
CPS	Combined positive scores
pMMR	Mismatch repair proficiency
CT	Computed tomography
AUC	Area under the curve
ECOG PS	Eastern Cooperative Oncology Group performance status score
preAintra	Intratumoral regions at baseline atrial phase
preVintra	Intratumoral regions at baseline venous phase
preAperi	Peritumoral regions at baseline atrial phase
preVperi	Peritumoral regions at baseline venous phase
postAintra	Intratumoral regions at follow-up atrial phase
postVintra	Intratumoral regions at follow-up venous phase
postAperi	Peritumoral regions at follow-up atrial phase
postVperi	Peritumoral regions at follow-up venous phase
ΔAintra	Changes between baseline and follow-up features of intratumoral regions at atrial phase
ΔVintra	Changes between baseline and follow-up features of intratumoral regions at venous phase
ΔAperi	Changes between baseline and follow-up features of peritumoral regions at atrial phase
ΔVperi	Changes between baseline and follow-up features of peritumoral regions at venous phase
IQR	Interquartile ranges
ICCs	Intraclass correlation coefficients
LASSO	Least absolute shrinkage and the selection operator
C-index	Harrell’s concordance index
CI	Confidence interval
HR	Hazard ratio
ΔVintra_ZV	ΔVintra_original_glszm_Zone Variance
postVperi_Sphericity	Postvperi_original_shape_Sphericity
NSCLC	Non–small cell lung cancer
